# Bi-Manual Examination in Children

**Published:** 1905-05-13

**Authors:** 


### BI-MANUAL EXAMINATION IN CHILDREN.

Dr. Geo. Carpenter has done excellent service in his various papers emphasising the value in the pelvic and abdominal diseases of children of the combination of rectal exploration with deep palpation applied through the abdominal wall. The relatively shallow measurement of the pelvic basin in early life permits ready and free investigation, not only of the contents of the pelvis, but also of the viscera in the lower abdomen. Whilst it may be advisable, as suggested, to anæsthetise the child in a doubtful diagnosis, or when there is present a definitely abnormal condition the nature of which has to be determined, it is well to remember that rectal examination in early life is very easily performed, and that it need cause little or no discomfort. Hence the practice, more particularly by those of junior experience, ought to be made a frequent one, as it is only by this measure that one can acquire the ability to distinguish confidently between the normal and the definitely abnormal. Such an aim is one of the defences of the exhortation to the habit of constant physical examination in other parts of the body, and the consideration applies equally to exploration through the rectum. Obviously, too, the same systematic method is necessary to the development of that delicacy of touch which, like other sense developments, needs repeated practice for its effective cultivation. This applies to the case of adults as well as to children, and there can be no doubt that were the dictum of a well-known professor of surgery in which students are urged never to miss a chance of rectal examination generally remembered, not a few blunders in diagnosis would be avoided. Perhaps no experience points this moral more emphatically than that gained in dealing with cases of appendicitis, in a certain proportion of which, though nothing can be confidently determined through the abdominal wall, rectal examination at once detects an appreciable tumour.

